# Supplementary material for: Why, what and how do European healthcare managers use performance data? Results of a survey and workshop among members of the European Hospital and Healthcare Federation
Source: PLoS One. 2020 Apr 8;15(4):e0231345. doi: 10.1371/journal.pone.0231345 (PMC7141666; doi:10.1371/journal.pone.0231345)
Supplement: S1 Appendix — The final version of the online questionnaire. (DOCX) [file pone.0231345.s001.docx]

S1 Appendix: “Online questionnaire”

**Use of performance data for “evidence-informed decision-making in healthcare management” – a survey among HOPE Exchange Programme participants and local hosts**

Thank you for taking the time to participate in this survey.

Completing the questionnaire should not take more than 15 minutes of your time.

What do we mean by *performance data*?

Managerial work in healthcare is increasingly focused on delivering value, also known as a “value-based healthcare” approach. In order to manage for value, contributing to the health of individuals and populations, we need to know what the desired outcomes are. We also need to know how to reach them and with which resources. For all of this, we need evidence in the form of available, reliable and actionable data. This is what we mean by terms *performance data, information and intelligence* in this questionnaire.

How does this link to your HOPE Exchange Programme participation?

In line with the 2019 HOPE Exchange Programme overarching topic of “Evidence-informed decision-making in healthcare management”, the purpose of this questionnaire is to learn more about the use of performance data in hospitals and other healthcare organisations around Europe.

How is this questionnaire structured?

The questionnaire consists of four sections. In the first part, you will be asked questions about your background as a manager. Next two sections are on the use of performance data in your organisation and in your daily managerial work. The final part of the questionnaire will provide you with a list of statements regarding the use of performance data. We would like to know to what extent do you agree with each of the statements.

*A note on privacy*

*HOPE Exchange Programme participants and local hosts are invited to complete the questionnaire. Email communication will go through the HOPE office in Brussels, supported by researchers from the HealthPros project at the Amsterdam University Medical Centers (Amsterdam UMC), location AMC, University of Amsterdam and funded through the Marie-Curie Innovative Training Network HealthPros.*

*The data collected with this questionnaire will only be analysed and used by the HealthPros project researchers to reach the research objectives described above. Data will never be disclosed to any external party (except authorised third parties such as legal authorities). Data will be stored in compliance with standards of security and confidentiality as applied in the Amsterdam UMC’s Clinical Research Unit facilities. Anonymised data will be stored for five years after the last day of the HealthPros project (December 31^st^, 2021) for audit purposes. At the end of this period, the data will be erased. Amsterdam UMC’s Medical Ethics Committee (location AMC) assessed the research proposal and decided that it does not require an official approval.*

*If needed, please consult relevant persons in your organisation for approval to participate in this survey and provide information about your organisation.*

*You are entirely free to accept or refuse to participate to this survey. If you accept, you have the right to stop, at any time, without any justification. In accordance with the General Data Protection Regulation (Regulation (EU) 2016/679 of the European Parliament), you have a right of access, rectification and erasure of your personal data. If you want to exercise any of these rights, please contact AMC’s Data Privacy Officer at* [*fg@amc.nl*](mailto:fg@amc.nl)*.*

*If you, for any reason, wish to contact the survey administrators, please do so by sending an email to* [*d.ivankovic@amc.nl*](mailto:d.ivankovic@amc.nl)*.*

Question group A: **Your background as a manager**

In this section we want to get to know a little bit more about you – where you work and what are your managerial roles and responsibilities.

If you work for multiple organisations, please answer the questionnaire for the organisation that you spend most of your time at.

| Q. Code | Question type  (Format) | Question  (On screen text) | Answers  (Options and values) | Comment(s), conditions and help text |
| --- | --- | --- | --- | --- |
| A1 | Short free text | **Please provide your full name.** |  |  |
| A2 | Single choice (radio) | **What is your gender?** | 1 = Male  2 = Female  3 = Other / prefer not to specify |  |
| A3 | Single choice (radio) | **Do you agree to be informed (via e-mail) about the results of this survey in the following months?** | 1 = Yes  2 = No |  |
| A4 | Short free text | **Please provide your e-mail address.** |  | Condition: If A3 == “Yes” / 1  Validation: /^.+?@.+?\..+$/ |
| The next set of questions is about the organisation that you work for.  If you work for multiple organisations, please answer the questionnaire for the organisation that you spend most of your time at. | | | | |
| A5 | List (dropdown) | **In which country do you work?** |  | <https://www.britannica.com/topic/list-of-countries-1993160> |
| A6 | Single choice (radio) | **What best describes the organisation that you work for?** | 1 = Hospital – smaller local  2 = Hospital – larger regional / teaching  3 = Hospital – university  4 = Long-term care  5 = Mental care  6 = Primary care  7 = Central government (ministry, government agencies)  8 = Local and regional government  X = Other | Option “Other” == “Yes” |
| A7 | Single choice (radio) | **What is the ownership / economic profile of the organisation that you work for?** | 1 = Public  2 = Private not-for-profit  3 = Private for-profit  X = Other | Option “Other” == “Yes” |
| A8 | Short free text | **What is the name of the organisation that you work for?** |  |  |
| The following questions ask about your managerial roles, responsibilities and competencies. | | | | |
| A9 | Short free text | **What is your position / job description?** |  |  |
| A10 | Single choice (radio) | **Which one of the following statements best describes your management role?** | 1 = I mostly manage plans and strategies for the whole organisation  2 = I mostly manage care processes  3 = I mostly manage the support of care processes (e.g. human resources, quality, IT, financing…)  4 = Not applicable / Not a manager |  |
| A11 | Array | **How much do these characteristics describe you as a manager?** | Subquestions:  A11.1: Experience / past results  A11.2: Knowledge / training  A11.3 Social skills / influence  ---  Answers:  4 = A great deal  3 = Considerably  2 = Moderately  1 = Slightly  0 = Not at all | Condition: If A10 <> “Not applicable / Not a manager” |
| A12 | Single choice (radio) | **If you are directly managing people, how large is the group of people that you are responsible for?** | 1 = 1-5  2 = 6-20  3 = 21-50  4 = 51-200  5 = More than 200  6 = Not applicable | Condition: If A10 <> “Not applicable / Not a manager” / 4 |
| A13 | Single choice (radio) | **How many years of relevant managerial experience do you have?** | 1 = Less than 5  2 = 5-10  3 = 11-20  4 = More than 20  5 = Not applicable | Condition: If A10 <> “Not applicable / Not a manager” / 4 |
| This, last set of questions, is about your HOPE Exchange Programme participation. | | | | |
| A14 | Single choice (radio) | **When did you participate in the HOPE Exchange Programme?** | 1 = 2015 and earlier  2 = 2016  3 = 2017  4 = 2018  5 = 2019 / Will participate this year  6 = Did not participate |  |
| A15 | List (dropdown) | **Where did you do (or will do) your HOPE Exchange?** |  | List of European countries. |

Question group B: **Use of performance data in your organisation**

In this section, we want to learn more about your organisation. How is performance data collected, reported and used? We are also interested in data sources as well as reporting and benchmarking tools used.

Please try to answer questions in this section from the perspective of your organisation as a whole.

First set of questions in this section is based on the WHO PATH model*, which defines dimensions in which performance data can be collected, reported and used.

Performance *data* are raw measurements before any analysis. Once these have been analysed or processed, they become performance *information*. Performance *intelligence* is a compilation of performance information for users that may have different usage purposes such as strategic or operational decision-making.

In the following table, we present examples of performance data -> information -> intelligence in the PATH model:

| Performance data  (Dimensions) | Performance information  (Examples) | Performance intelligence  (Examples) | |
| --- | --- | --- | --- |
| Clinical effectiveness | - Mortality rates - Readmission rates - Day surgery for cataract / knee arthroscopy / inguinal hernia / tonsillectomy / cholecystectomy | 30-day readmission rates metric included in the key performance indicator (KPI) dashboard. Also showing historical trend data (change over time) through traffic-light system symbols:   - Red – rate worsening; - Orange – no change; - Green – rate improving. | |
| Efficiency | - Length of stay - Surgical theatre use - Costs of antibiotics per patient - Cash-flow / debt ratio |  |  |
| Safety  (both patients and staff) | - Prophylactic antibiotic use - Staff needle injuries | A yearly report on staff safety showing needle stick injuries rate with comparison among clinical wards, analysis and interpretation of differences and recommendations for improvement. | |
| Patient centredness | - Patient survey score on access to care - Patient survey score on amenities of care |  |  |
| - Collecting | - **Reporting** | | - **Using** |

*PATH is a performance assessment system designed by the World Health Organization to support hospitals in defining quality improvement strategies, questioning their own results and translating them into actions for improvement
[**http://www.pathqualityproject.eu/what_is_path.html**](http://www.pathqualityproject.eu/what_is_path.html).

| Q. Code | Question type  (Format) | Question  (On screen text) | Answers  (Options and values) | Comment(s), conditions and help text |
| --- | --- | --- | --- | --- |
| B1 | Array | **How much does your organisation collect performance data for the following dimensions?** | Subquestions:  B1.1: Clinical effectiveness  B1.2: Efficiency  B1.3: Safety (patients and staff)  B1.4: Patient centredness  --- Answers:  4 = A great deal  3 = Considerably  2 = Moderately  1 = Slightly  0 = Not at all  X = Do not know |  |
| B2 | Array | **How much does your organisation report performance data for the following dimensions?** | Subquestions:  B2.1: Clinical effectiveness  B2.2: Efficiency  B2.3: Safety (patients and staff)  B2.4: Patient centredness  --- Answers:  4 = A great deal  3 = Considerably  2 = Moderately  1 = Slightly  0 = Not at all  X = Do not know |  |
| B3 | Array | **For decision-making, how much does your organisation use performance data from the following dimensions?** | Subquestions:  B3.1: Clinical effectiveness  B3.2: Efficiency  B3.3: Safety (patients and staff)  B3.4: Patient centredness  --- Answers:  4 = A great deal  3 = Considerably  2 = Moderately  1 = Slightly  0 = Not at all  X = Do not know |  |
| The following questions are about methods and tools used to report and use performance data in your organisation. | | | | |
| B4 | Multiple choice | **Which methods and tools are used to report performance data in your organisation?** | 1 = Verbal communication  2 = Written reports  3 = Score cards  4 = Dashboards  5 = Control charts  6 = None  X = Other | Option “Other” == “Yes”  Exclusive option == "None" / 6  Answers 2 - 5 (Written reports;  Score cards;  Dashboards;  Control charts) additionally represented with images. |
| B5 | Long free text | **Please provide an example of the methods and tools used.**  If you feel more comfortable providing an example in your own language, feel free to do so. |  | Condition: If B4 <> “None” / 6 |
| B6 | Single choice (radio) | **How much is performance data from your organisation used to benchmark with other comparable organisations?** | 4 = A great deal  3 = Considerably  2 = Moderately  1 = Slightly  0 = Not at all  X = Do not know |  |
| B7 | Long free text | **Please provide an example of using performance data for benchmarking, in your organisation.**  If you feel more comfortable providing an example in your own language, feel free to do so. |  | Condition:  IF B6 = “A great deal” / 4 OR B6 = “Considerably” / 3 OR B6 = “Moderately” / 2 OR B6 = “Slightly” / 1 |
| B8 | Array | **How much are the following data sources used as performance data in your organisation?** | Subquestions:  B8.1: Electronic health records  B8.2: Administrative databases  B8.3: Population survey data  B8.4: Patient reported data (e.g. PREMs and PROMs)  B8.5: Disease/condition-based registries  B8.6: Population-based registries  B8.7: Third-party assessments (e.g. accreditation)  B8.8: Regulatory inspection data  ---  Answers:  4 = A great deal  3 = Considerably  2 = Moderately  1 = Slightly  0 = Not at all  X = Do not know |  |
| B9 | Array | **How important are the following purposes to collect and report performance data in your organisation?** | Subquestions:  B9.1: Internal quality assurance  B9.2: Internal quality improvement  B9.3: External accountability  B9.4: External comparison/ benchmarking  ---  Answers:  4 = Very important  3 = Important  2 = Moderately important  1 = Slightly important  0 = Not important  X = Do not know | Help text:  Internal quality assurance = Making sure that internally set standards and goals are achieved.  Internal quality improvement = Improving performance regardless of achieving the goals or not.  External accountability = Making sure that externally set standards and goals are achieved.  External comparison / benchmarking = Comparing to others in a standardised way. |

Question group C: **Using performance data in your daily work**

In this section, we would like to find out more about the use of performance data and intelligence in your daily work.

Please try to answer this set of questions from the perspective of your daily managerial work in your unit/team.

| Q. Code | Question type  (Format) | Question  (On screen text) | Answers  (Options and values) | Comment(s), conditions and help text |
| --- | --- | --- | --- | --- |
| C1 | Single choice | **In your daily work, how much do you participate in collecting performance data?** | 4 = A great deal  3 = Considerably  2 = Moderately  1 = Slightly  0 = Not at all |  |
| C2 | Long free text | **Please provide an example of your work in collecting performance data.**  If you feel more comfortable providing an example in your own language, feel free to do so. |  | If C1 <> “Not at all” / 0 |
| C3 | Long free text | **In your daily work, how much do you participate in preparing reports based on performance data?** | 4 = A great deal  3 = Considerably  2 = Moderately  1 = Slightly  0 = Not at all |  |
| C4 | Long free text | **Please provide an example of your work in preparing reports based on performance data.**  If you feel more comfortable providing an example in your own language, feel free to do so. |  | If C3 <> “Not at all” / 0 |
| C5 | Array | **How important are the following purposes to collect and report performance data in your daily work?** | Subquestions:  C5.1: Internal quality assurance  C5.2: Internal quality improvement  C5.3: External accountability  C5.4: External comparison/ benchmarking  ---  Answers:  4 = Very important  3 = Important  2 = Moderately important  1 = Slightly important  0 = Not important | Help text:  Internal quality assurance = Making sure that internally set standards and goals are achieved.  Internal quality improvement = Improving performance regardless of achieving the goals or not.  External accountability = Making sure that externally set standards and goals are achieved.  External comparison / benchmarking = Comparing to others in a standardised way. |
| C6 | Single choice | **In your daily work, how much do you use performance data for decision-making?** | 4 = A great deal  3 = Considerably  2 = Moderately  1 = Slightly  0 = Not at all |  |
| C7 | Long free text | **Please provide an example of using performance data for decision-making in your daily work.**  If you feel more comfortable providing an example in your own language, feel free to do so. |  | If C6 <> “Not at all” / 0 |

Question group D: **Enablers and barriers to the use of performance data**

In this section we want to find out more about your experiences and opinions on the use and usefulness of performance data in your work.

*Some questions and statements are based on previous research by the Canadian Institute for Health Information (CIHI) and New England Journal of Medicine’s (NEJM) Catalyst project.*

| Q. Code | Question type  (Format) | Question  (On screen text) | Answers  (Options and values) | Comment(s), conditions and help text |
| --- | --- | --- | --- | --- |
| D1 | Array | **How effective do you consider your organisation’s use of data for each of the following?** | Subquestions:  D1.1 = Guiding business leadership  D1.2 = Guiding clinical leadership  D1.3 = Guiding population efforts  D1.4 = Supporting care decisions for individual patients  ---  Answers:  4 = Extremely effective  3 = Very effective  2 = Effective  1 = Not very effective  0 = Not at all effective  X = Not applicable |  |

| Q. Code | Question type  (Format) | Question  (On screen text) | Answers  (Options and values) | Comment(s), conditions and help text |
| --- | --- | --- | --- | --- |
| D2 – D13 | List (radio) | **How much do you agree with each of the following statements?** | 4 = Completely  3 = Considerably  2 = Moderately  1 = Slightly  0 = Not at all  X = Do not know | Display columns == “7” |

D2: I feel that a large amount of data is currently collected but very little is used.

D3: I am confident about the reliability of performance data in my organisation.

D4: I see data being manipulated and therefore do not trust performance intelligence that results from this data.

D5: I think that the performance indicators which I use in my work are valid.

D6: I am involved in the development and selection of performance indicators to be used in my daily work.

D7: I think that external reporting on my team’s (or unit’s) performance is helping us improve.

D8: I feel that the performance and accountability reporting requirements are increasing rapidly without a corresponding increase in budgets to allow for this.

D9: I feel confident in my skills of using data, indicators and scorecards to monitor performance.

D10: I see that smaller hospitals have the same reporting requirement as larger ones, but given their financial means do not have the same infrastructure nor manpower to focus on performance and accountability reporting.

D11: I think that funding only permits us to capture and report the data but we don't have staff with the needed time and knowledge to analyse the data.

D12: I feel that, thanks to performance intelligence, decisions taken in my daily work are more evidence-informed.

D13: I feel that decision-making based on performance data makes it easier for me as a manager to explain and justify my decision.
