# Supplementary figures and images for: Why, what and how do European healthcare managers use performance data? Results of a survey and workshop among members of the European Hospital and Healthcare Federation
Source: PLoS One. 2020 Apr 8;15(4):e0231345. doi: 10.1371/journal.pone.0231345 (PMC7141666; doi:10.1371/journal.pone.0231345)

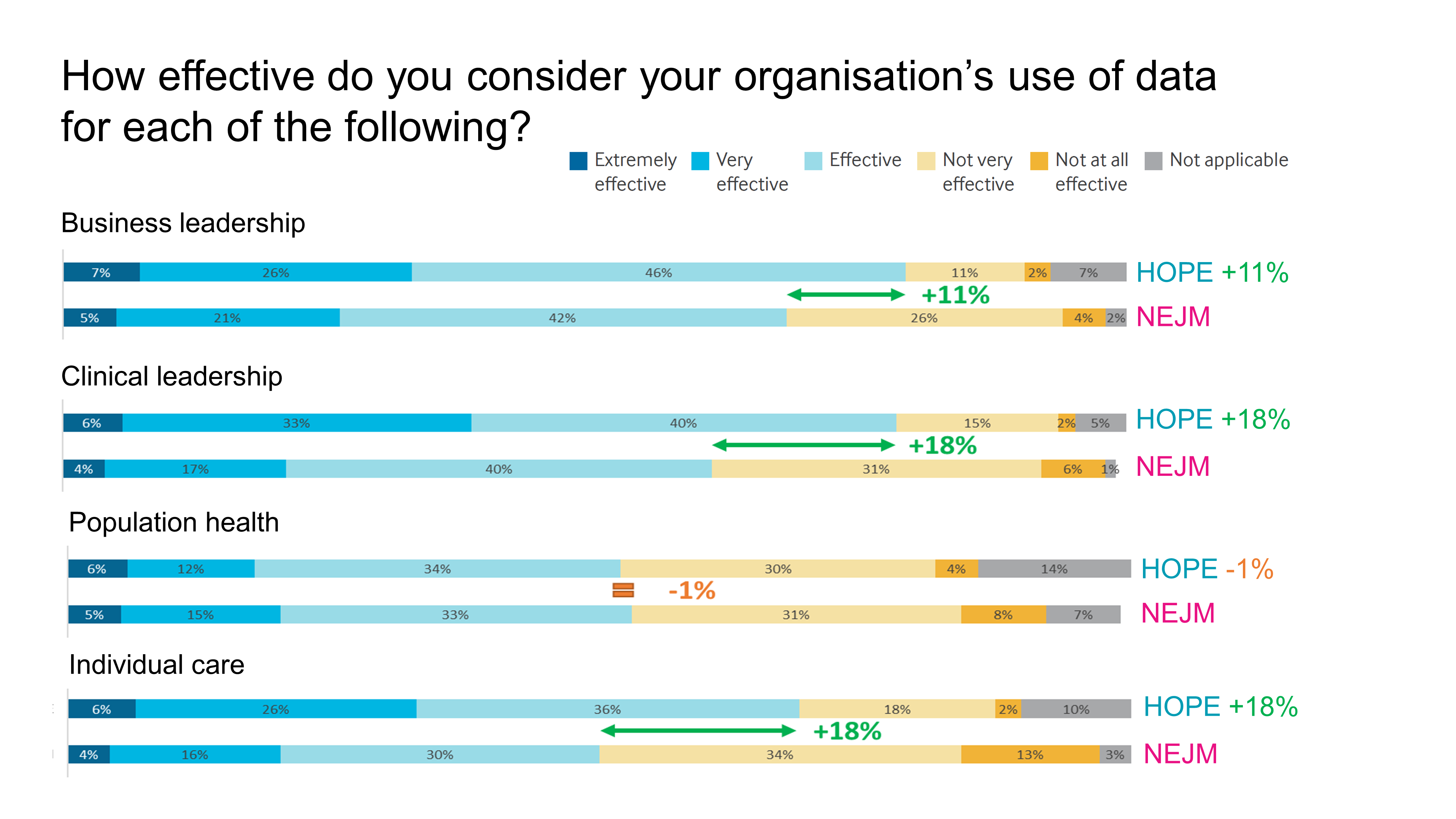

Supplement: S1 Fig — Comparison between the results to the same question used for the NEJM Catalyst survey among the US healthcare managers and European healthcare managers in this survey. (TIF) [file pone.0231345.s003.tif]
